# Role of MRI in uterine didelphys with co-existing endometrial carcinosarcoma

**DOI:** 10.1259/bjrcr.20180010

**Published:** 2018-10-31

**Authors:** Jill McGregor, Gillian Thompson

**Affiliations:** 1 Emergency Medicine Department, Ulster Hospital Dundonald, Belfast, UK; 2 Imaging Department, Western Health and Social Care Trust, Londonderry, UK

## Abstract

A case report reviewing MRI in a patient diagnosed with carcinosarcoma and uterine didelphys following presentation with post-menopausal bleeding. Staging MRI images demonstrate the anatomical anomaly and pathological features in these co-existing conditions. This report presents an interesting case of carcinosarcoma in a uterine didelphys. This anatomical abnormality is an uncommon finding and is very rarely complicated by carcinosarcoma. There are very few cases reported to date of this unusual condition. Our case is of a 77-year-old female, para 9, who presented with post-menopausal bleeding for 1 month. She followed the pathway for endometrial cancer using Northern Ireland Cancer Network clinical guidelines. This female’s co-existing uterine anomaly and malignant pathology are outlined, staged and beautifully illustrated with dedicated pelvic MRI. The images captured by MRI are used in all aspects of the patients care and treatment planning, and show the benefit of this modality in multidisciplinary meetings guiding gynaecological-oncology surgeons, who must aware of the anatomical variants before embarking on definitive surgery.

## BACKGROUND

Uterine didelphys is an uncommon finding and is very rarely complicated by carcinosarcoma. There are few cases reported to date of these conditions co-existing. The rare anomaly of didelphys uterus is estimated in 1/3000 females.^1^ Fibroids and benign tumours are relatively common findings in uterine didelphys; however there are few recorded cases of sarcoma, the malignant mesenchymal tumour.^2^ A literature search was carried out of didelphys uterus and carcinosarcoma and there appears to be only two other cases similar to ours described.

Risk factors for endometrial cancer include oestrogen replacement, obesity, polycystic ovarian syndrome, null parity, tamoxifen and diabetes mellitus.^3^ The most common presentation is post-menopausal bleeding, as is the case with our patient.

## CLINICAL PRESENTATION

A 77-year-old female (para 9) presented with post-menopausal bleeding for 1 month. She has had three sets of twins by caesarean section.

Past medical history included myocardial infarction, primary coronary intervention with stent insertion, hypertension and hypercholesterolemia.

On examination, clinical obesity was noted and bimanual palpation did not reveal a pelvic mass. On speculum examination, two cervices were identified.

## DIFFERENTIAL DIAGNOSIS

The differential diagnosis for post-menopausal bleeding includes endometrial atrophy, endometrial polyps, submucosal fibroids, endometrial hyperplasia, endometrial carcinoma (approx. 10%) and oestrogen withdrawal.^4^

## INVESTIGATION/IMAGING FINDINGS

Initially she underwent trans-vaginal ultrasound scan at gynaecology outpatient clinic, which was diagnostic for uterine didelphys, with two distinct endometrial cavities and separation of the uterine fundi. The left endometrium measured 2 mm (within normal limits for post-menopausal female) and the right endometrium measured 10 mm, raising the suspicion of endometrial pathology.

The patient then proceeded to outpatient hysteroscopy which revealed stenosis of the left cervix. The right cervix and cavity showed a vascular suspicious appearing endometrium, which was subsequently biopsied. It was not possible to sample the left endometrium due to the cervical stenosis.

Pathology report revealed fragments of carcinosarcoma (malignant mixed mullerian tumour - MMMT), with automatic grading of Grade III endometrial carcinoma.

Following this, the female was discussed at local gynaecological-oncology multidisciplinary meeting and after clinic review to discuss the diagnosis, she proceeded to staging MRI scan of the gynaecological pelvis.

Care pathway for endometrial cancer was followed using Northern Ireland Cancer Network (NICaN) clinical guidelines—within 28 days patients should undergo ultrasound scan, hysteroscopy, patient consultation to discuss diagnosis before MRI and discussion at multidisciplinary team meeting with a decision to treat being made. This female had imaging using an Magnetom Aera 1.5T MRI scanner with sequence protocol as per NICaN imaging guidelines using Gadovist 7.5 ml (Bayer) (Table 1).

**Table 1.** NICaN imaging guidelines

CT	**Area scanned**	**Oral contrast**	**IV contrast vol/sec**	**Delay(s)**	**Max slice thickness**	**Notes**
	abdomen and pelvis	1 Litre 2% gastrogaffin	100 ml at 3 ml sec^-1^	70 s portovenous phase	5 mm reformat from 1.25 mm slices (32 slice MDCT)	
MRI	**Area**	**Sequence**		**Plane**	**Slice Thk**	**Notes**
	Abdomen and pelvis	T1W		Axial LFOV	Case dependnt	At least cover from renal hilum down
		T2W		Axial LFOV		
	Pelvis	T2W		Sag SFOV		
		T2W		Obl axial SFOV		Small, oblique, perpendicular to the cervix/uterus
		± TIWGE		Obl axial SFOV pre-contrast		
		± TIWGE		Obl axial SFOV post-contrast		
		T2W		Axial LFOV abdomen		20 s delay
PET	PET is not useful in endometrial carcinoma					

LFOV, long field of view; PET, positron emission tomography; SFOV, short field of view.; T1W, *T*_1_ weighted; T1W, *T*_1_ weighted.

Northern Ireland Cancer Network imaging guidelines for histologically proven endometrial cancer. These guidelines help to standardise the imaging modalities and protocols used therefore making regional multidisciplinary meetings more uniform.

The MRI demonstrated abnormal widening of the right endometrial cavity with invasion into the myometrium beyond the junctional zone. The pathological signal extended from the uterine fundus to the internal os without cervical stromal involvement.

No parametrial or adnexal extension shown on post-contrast sequences. No significant iliac or para-aortic lymphadenopathy identified.

**Figure 1.**
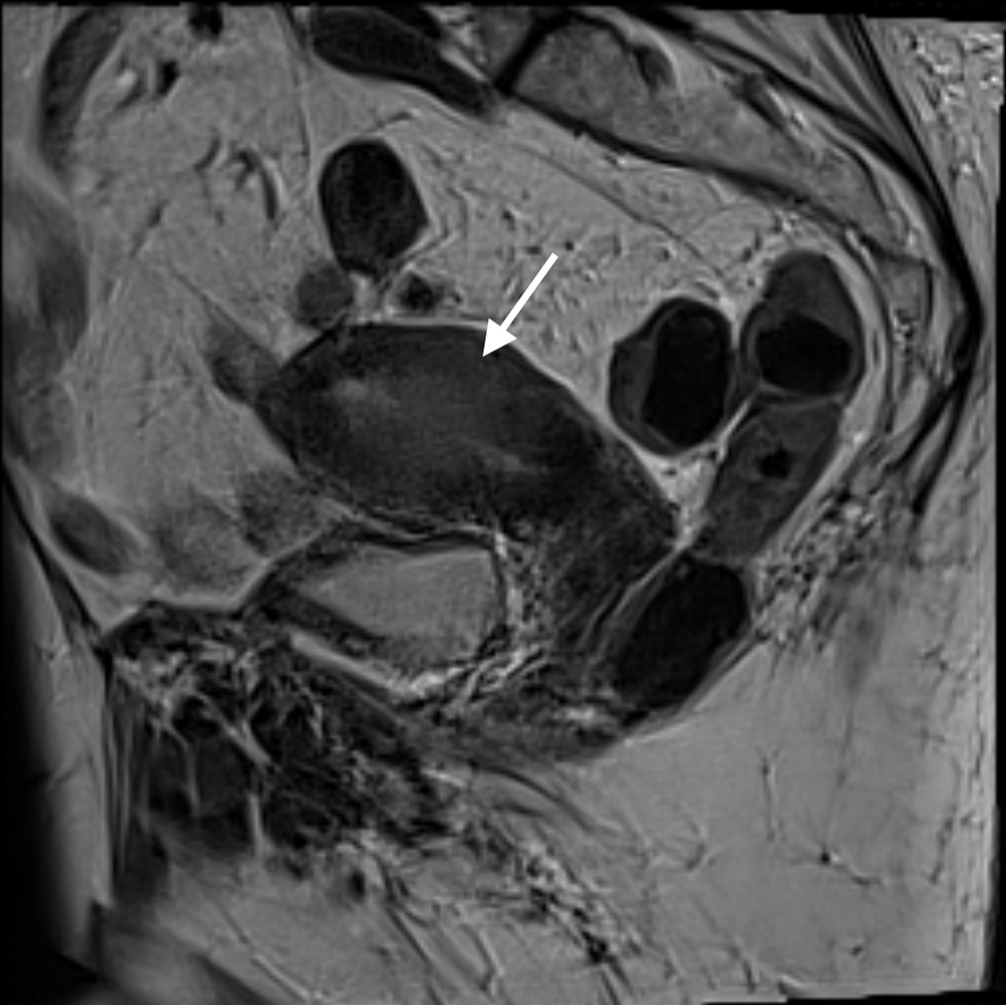
MRI *T*_2_ sagittal image illustrating thickened endometrium and myometrial invasion (see arrow) within the right uterus.

**Figure 2.**
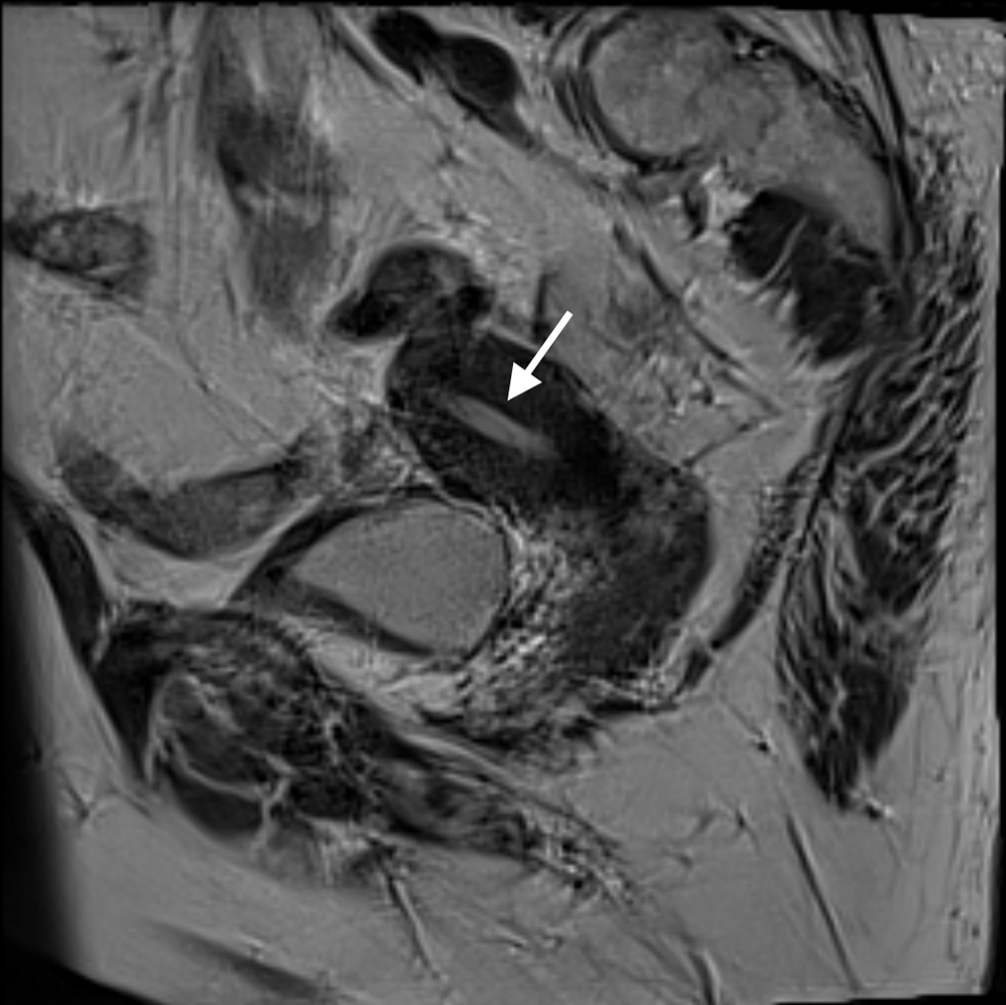
MRI *T*_2_ sagittal image demonstrating left uterus with normal dimensions of the post-menopausal endometrium (see arrow). Compare with Figure 1 above.

**Figure 3.**
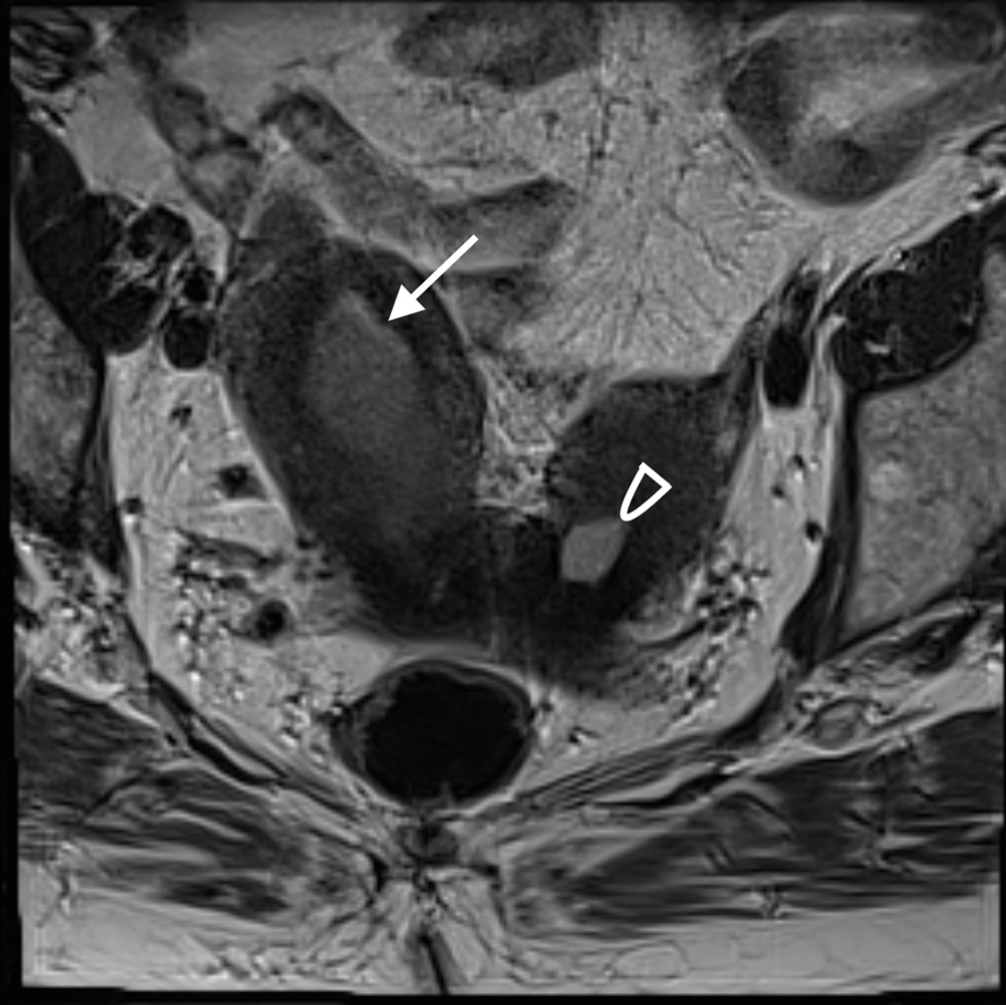
MRI *T*_2_ coronal oblique image illustrating didelphys uterus with two uterine fundi. The right endometrial cavity (see arrow) is distended with pathologically proven carcinosarcoma, compared to the normal post-menopausal left endometrial cavity (see arrowhead).

**Figure 4.**
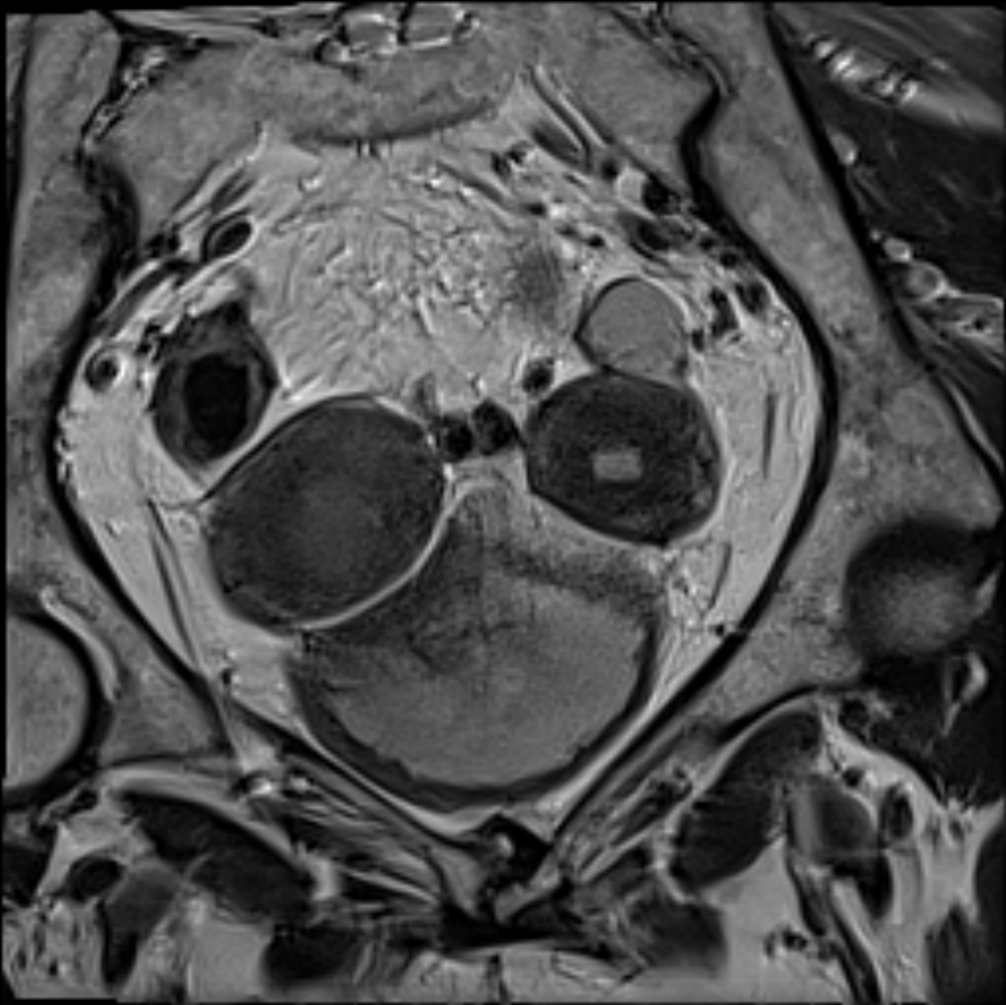
MRI *T*_2_ axial oblique image showing the abnormal intermediate signal tumour occupying the right endometrial cavity and invading the adjacent myometrium.

**Figure 5.**
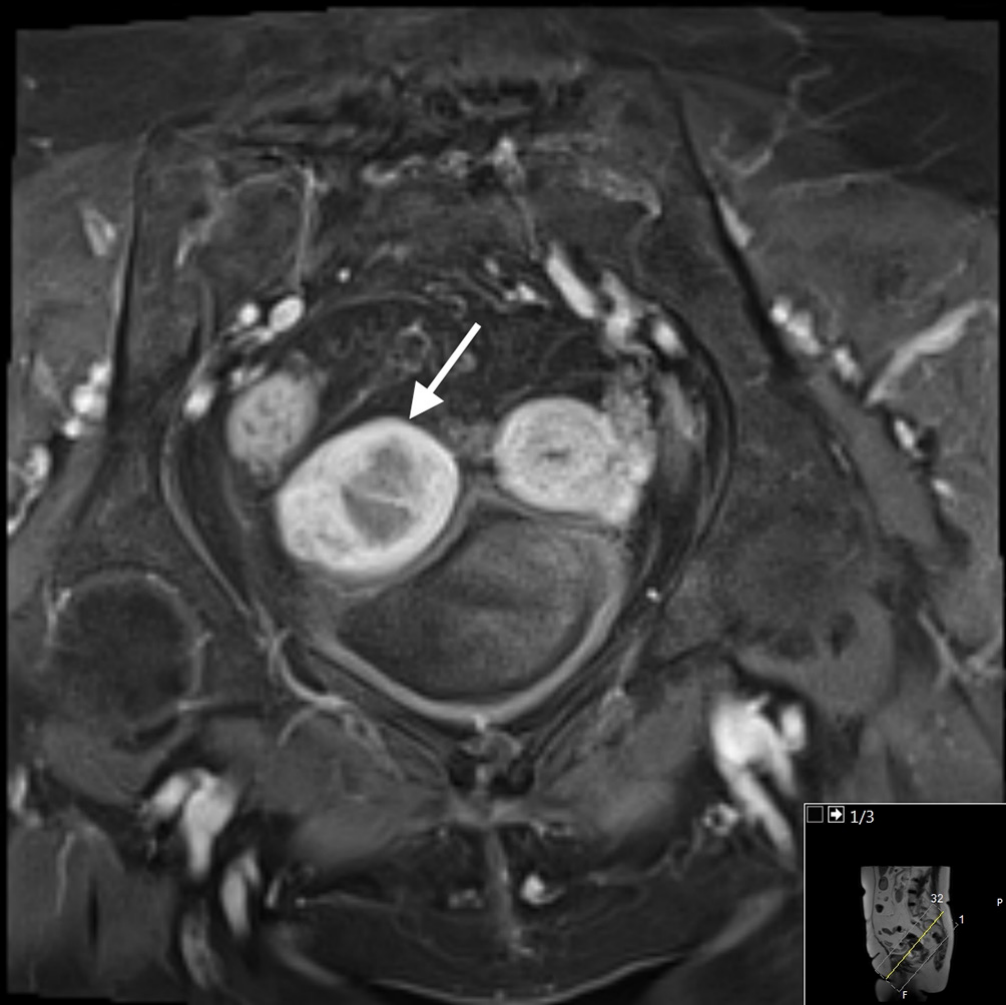
MRI *T*_1_ starvibe axial oblique post-gadolinium image showing the non-enhancing endometrial tumour with myometrial invasion (see arrow), in comparison with the normal enhancing myometrium within the left uterus.

The left uterus, endometrial cavity and cervix had a normal MRI appearance for a post-menopausal patient. The International Federation of Gynaecology and Obstetrics (FIGO) MRI staging of the right uterus was Stage 1b (>50% myometrial invasion); node negative (Figures 1–5 and Table 2)*.*

**Table 2.** The International Federation of Gynaecology and Obstetrics MRI staging for endometrial cancer

**Stage I**	Tumour confined to the uterus
IA	<50% invasion of the myometrium
IB	≥50% invasion of the myometrium
**Stage II**	Tumour invades the cervical stroma but does not extend beyond the uterus
**Stage III**	Local or regional spread of tumour
IIIA	Serosal or adnexal invasion
IIIB	Vaginal or parametrial involvement
IIIC IIIC1 IIIC2	Metastasis to pelvic or para-aortic lymph nodes Pelvic lymph node involvement Para-aortic lymph node involvement (with or without pelvic nodes)
**Stage IV**	Extension to the pelvic wall, lower one-third of the vagina, or hydronephrosis or non-functioning kidnety
IVA	Invasion of bladder or bowel mucosa
IVB	Distant metastases, including abdominal, or involvement of inguinal lymph nodes

**Figure 6.**
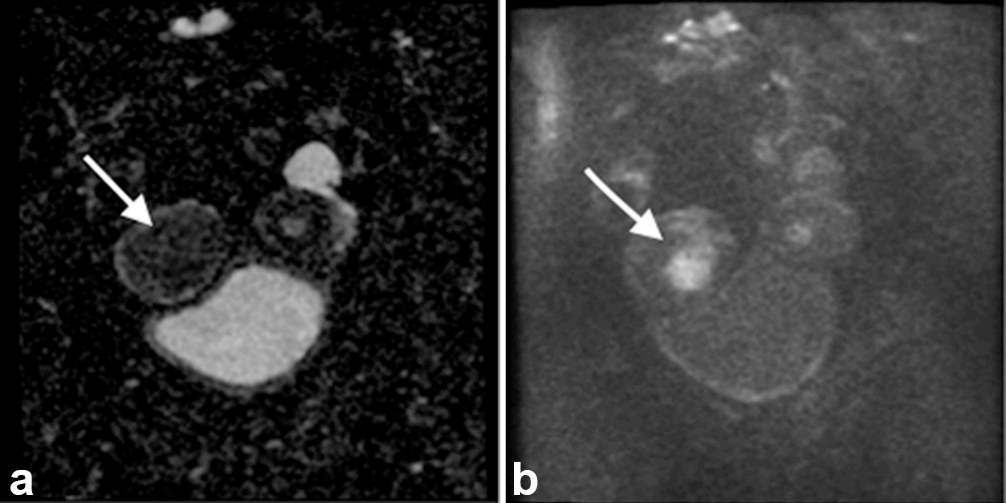
(a) MRI diffusion-weighted images. Apparent diffusion coefficient map (b) b800 reference image showing diffusion restriction pattern confirming the likely malignant nature of the right uterine tumour (see arrow).

Diffusion-weighted images were also obtained since they provide a useful adjunct to the diagnostic information^5^ provided by the regionally recommended sequences (Figure 6a,b).

Due to the high grade pathology of this tumour, a volume acquisition computerised tomography scan from thoracic inlet to pubic symphysis was performed following intravenous contrast. This demonstrated no significant lymphadenopathy or evidence of distant metastatic spread.

## TREATMENT

Following staging and regional multidisciplinary team discussion, this female underwent surgery, which included laparoscopically assisted vaginal hysterectomy and bilateral salpingo-oophrectomy. She was deemed unfit for pelvic node dissection. Post-operative complication of a heavy per-vaginum bleed proceeded to a further post-operative CT showing vault haematoma.

Pathology from surgical specimen showed FIGO stage IIIa due to tumour within blood vessels of parametrium and right ovary. There was myometrial invasion of >50% with extensive lymphovascular permeation.

She proceeded to adjuvant radiotherapy but was unfortunately not medically fit enough for palliative chemotherapy. She continues to have regular gynaecological-oncology follow up and is currently approximately 8 months post surgery.

## DISCUSSION

Anatomical uterine abnormalities such as our case of uterine didelphys may be diagnosed using multiple imaging modalities such as ultrasound, MRI, fluoroscopy and CT. Local uterine malignancy staging is most accurately performed with dedicated MRI protocol. Both this female’s uterine anomaly and malignant pathology are detailed, staged and beautifully illustrated with dedicated pelvic MRI.

Ultrasound scanning is widely available and is a useful first imaging technique in patients with post-menopausal bleeding; however MRI is superior in its ability to not only stage myometrial invasion in endometrial malignancy but also assess invasion into the adjacent structures such as cervix, bladder and rectum. Demonstration of potential nodal involvement is also of benefit.

As shown in this case, MRI provides excellent anatomical detail and characterisation of both normal anatomy and co-existing pathology. MRI is a non-invasive, safe (with accurate check-listing) and usually well tolerated method of imaging with excellent results and good pathological correlation in staging of gynaecological malignancies. This correlation between MRI and pathological FIGO staging is regularly audited in our department to maintain recommended standards.^6^

MRI is an essential part of the care pathway and management protocol for patients with uterine malignancy such as in this case of carcinoma.

The radiologist is then able to fully participate and enhance each patient’s multidisciplinary meeting discussion to guide appropriate treatment. In our case of uterine didelphys and in another anatomical anomalies it is essential for the surgeon to be aware of the relevant anatomy before embarking on definitive surgery. MRI images as shown clearly demonstrate the variants in this patient. MRI is particularly useful in distinguishing between different uterine malformations and is in most cases superior in this regard in comparison to ultrasound and hysterosalpingography.^7^ For example, without detail regarding the fundal wall and endometrial cavities, it can be difficult to distinguish between bicornuate and septate uterine anomalies.

Safety issues, patient compliance, poor renal function and movement artefact including bowel peristalsis may limit MRI staging. Image interpretation is also subject to limitations in spatial resolution. In this case report the difference in MRI and pathological FIGO staging (*i.e.* 1b versus IIIa) is perhaps explained by the inability of MRI to assess early lymphovascular invasion. Diffusion-weighted imaging is evolving as a useful technique in MRI for assessing tumour invasion. It is not currently part of our NICaN imaging guidelines but should be considered for future inclusion, perhaps precluding the need for gadolinium.

MRI report, including FIGO staging, along with pathological grading help provide the clinician and the patient with information regarding the planned treatment, risk stratification and likely prognosis.^8^ MRI can provide information with regard to myometrial invasion, cervical stromal involvement, serosal breech, adnexal and lymph node involvement, all of which are necessary to accurately stage any grade of endometrial cancer.^9^

In our case, this female had Grade III endometrial carcinoma, also referred to as endometrial carcinosarcoma or malignant mixed mullerian tumour. Endometrial carcinoma may also be graded as I or II, both of which carry a better prognosis^10^ than the high grade carcinosarcoma, which carries a poor prognosis of 40% three year survival.^11^ The prognosis is further reduced because of our patients medical co-morbidities she was unable to have the recommended pelvic node clearance at the time of pelvic surgery.

## LEARNING POINTS

1. MRI is the most useful imaging modality in staging uterine malignancies such as carcinosarcoma. In this case anatomy is also well outlined and MRI is an excellent imaging technique for defining Mullerian tract anomalies.
2. Patient care and treatment should be guided by gynaecological-oncology multidisciplinary meeting and review of staging MRI images.
3. It is essential for the surgeon to be aware of the relevant anatomy before embarking on definitive surgery. MRI images as shown clearly demonstrate the anatomical variants in this patient.
